# National and State Estimates of SELECT Trial Eligibility and Its Public Health Impact in the US

**DOI:** 10.1001/jamanetworkopen.2024.20105

**Published:** 2024-07-03

**Authors:** John Erhabor, Michael Khorsandi, Michael J. Blaha

**Affiliations:** 1 The Johns Hopkins University School of Medicine, Baltimore, Maryland

## Abstract

This cross-sectional study of US adults examines the geographical distribution of individuals eligible to participate in the Semaglutide Effects on Heart Disease and Stroke in Patients With Overweight or Obesity (SELECT) trial to estimate potential cardiovascular health impacts of implementing the trial findings at state and national levels.

## Introduction

In the US, approximately two-thirds of adults are classified as overweight or obese, conditions linked to increased risks of hypertension, type 2 diabetes, and cardiovascular disease (CVD).^1^ The SELECT (Semaglutide Effects on Heart Disease and Stroke in Patients with Overweight or Obesity) trial showed that a weight loss dose of semaglutide significantly reduces the incidence of major adverse CVD outcomes in nondiabetic individuals who are either overweight or obese.^2^ Building on research highlighting variations in the cardiometabolic disease burden across states,^3^ this study examines the potential impact of the SELECT trial nationally and by state.

## Methods

Using 2022 Behavioral Risk Factor Surveillance System (BRFSS) data from all 50 US states and the District of Columbia, we computed state-level age-standardized weighted prevalence for individuals meeting SELECT trial criteria (age 45 years or older, body mass index [BMI] of 27 or higher [calculated as weight in kilograms divided by height in meters squared], with preexisting CVD, without diabetes). This was then multiplied by age-specific segments of the 2022 US Census population to derive age-adjusted total estimates.^4^ The *svy* command was applied per BRFSS guidelines to manage the survey design complexity.^5^ Johns Hopkins University deemed our study exempt from institutional review board review and informed consent requirements because we used deidentified, publicly available data. This study followed the Strengthening the Reporting of Observational Studies in Epidemiology (STROBE) reporting guidelines.

## Results

The study included 18 875 SELECT-eligible individuals, with 10 409 (55.2%) being men, 12 518 (66.4%) aged 65 years or older, and a mean (SD) BMI of 32.4 (5.3). Our analyses showed a 5.6% national prevalence, representing approximately 7.9 million individuals meeting the SELECT trial criteria. Among these individuals, treatment with semaglutide could prevent about 190 000 cardiovascular events in 5 years (Table). By state, higher SELECT-eligibility rates were observed in Arkansas (weighted percentage, 8.0%), Alabama (7.7%), and West Virginia (7.6%), with California (880 000), Texas (617 000), and Florida (589 000) having the highest number of eligible individuals (Figure).

**Table.** Estimated Reduction in Cardiovascular Events From Semaglutide Use Based on SELECT Trial and BRFSS Data

Description	Calculation	Result, No.
US population meeting SELECT criteria^a^	NA	7 857 000
Baseline CV event rate over 3.3-y follow-up (placebo)^b^	0.08 × 7 857 000	628 560
Expected CV events with semaglutide (using observed RRR) over 3.3-y follow-up	628 560 × 0.80	502 848
CV events prevented by treatment over 3.3-y follow-up^c^	628 560 − 502 848	125 712
Estimated CV events prevented over 5 y (assuming linear extrapolation)	125 712 × 5 / 3.3	190 473

Abbreviations: BRFSS, Behavioral Risk Factor Surveillance System; CV, cardiovascular; RRR, relative risk reduction; SELECT, Semaglutide Effects on Heart Disease and Stroke in Patients with Overweight or Obesity.

^a^ Represents the estimated total number of individuals meeting the SELECT trial criteria.

^b^ Represents the observed 8.0% event rate in the placebo group from the SELECT trial.

^c^ Calculated using the hazard ratio of 0.80 (95% CI, 0.72-0.90) from the SELECT trial, indicating a 20% reduction in risk compared with placebo.

**Figure.**
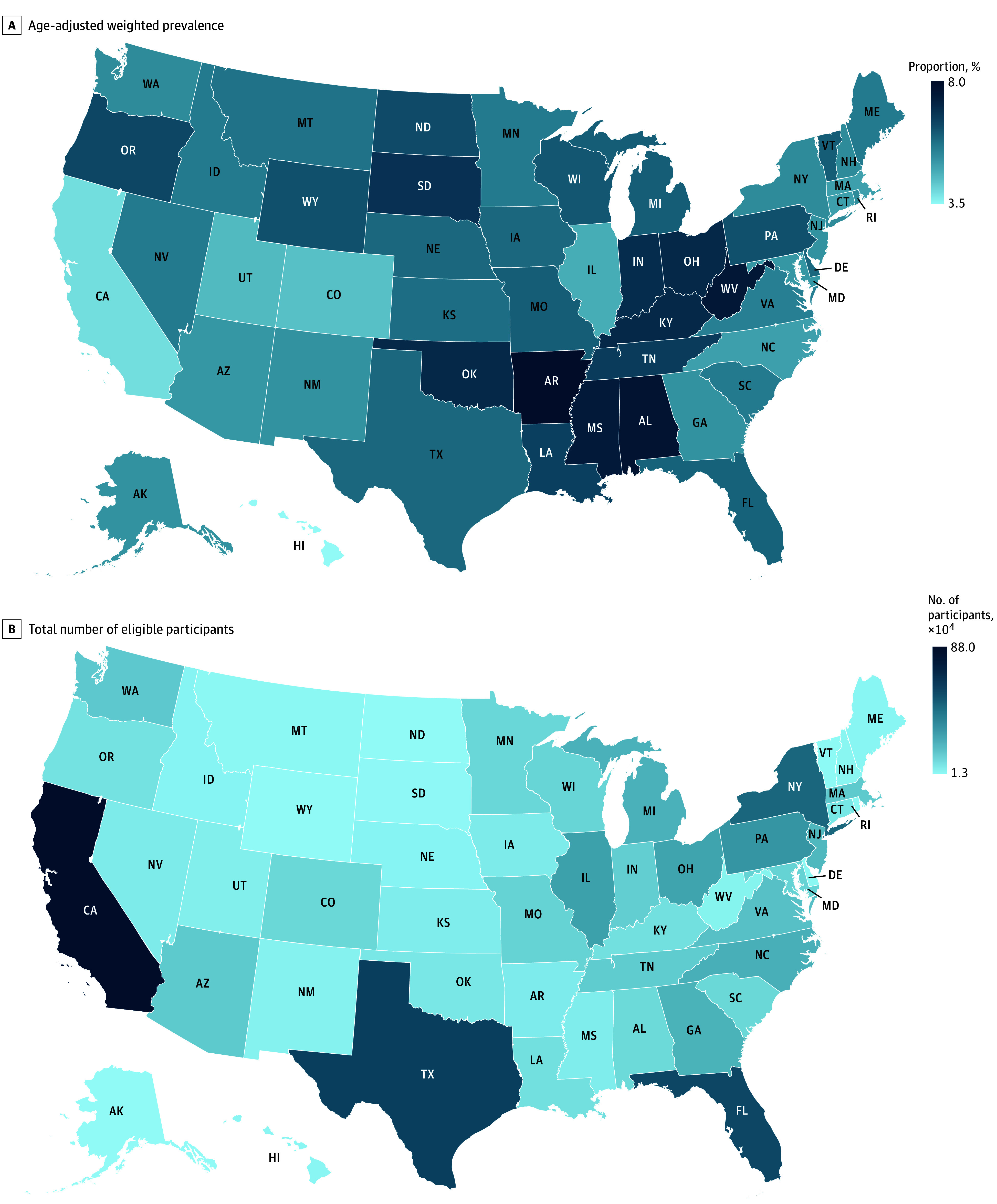
Age-Adjusted Weighted Prevalence and Total Number of Eligible Participants Across US States in the SELECT Trial

## Discussion

Our study extends prior work by examining the impact of implementing SELECT trial findings at national and state levels,^6^ improving understanding of regional variations in cardiometabolic disease burden and potential health care impact on state-level resources. These findings stress the extensive scope and implications of the SELECT trial when broadly applied to clinical practice, particularly given the high prevalence of CVD and obesity in the US. The potential for semaglutide to improve cardiovascular prognosis is significant, albeit costly. For example, if each eligible patient received semaglutide at a retail price of $16 188 per year, the total cost would amount to $127 billion per year. Potentially less costly alternatives for reducing obesity and CVD risk include lifestyle counseling, dietary fasting, and alternative pharmacological weight loss approaches. However, evidence supporting these measures is mixed, and long-term studies are needed to clarify their efficacy.

Despite the high costs, integrating semaglutide into the health care system could lead to significant additional benefits, including fewer cardiovascular hospitalizations, reduced need for invasive procedures, and enhanced patient quality of life. These improvements contribute to health care system efficiency by alleviating resource burdens and improving outcomes. Extending this treatment to all patients with obesity, including those with diabetes, could substantially improve overall quality of life by lowering cardiovascular risks, enhancing glycemic control, and potentially benefiting individuals with microvascular diseases, particularly nephropathy. However, managing adverse effects at scale, including gastrointestinal symptoms and potential cholecystitis, would be difficult but crucial for patient adherence and maximizing benefits.

Some limitations of this study include recall bias from self-reported data and data constraints preventing complete application of all SELECT trial exclusion criteria, which might lead to overrepresentation of certain factors such as history of planned revascularization and chronic pancreatitis. However, critical clinical inclusion criteria were thoroughly considered.

This study highlights the significant potential of the SELECT trial’s findings to improve public health outcomes nationwide or statewide through widespread semaglutide treatment. It also emphasizes the importance of strategic policy planning to tackle financial implications and regional disparities in treatment access. Policymakers are encouraged to incorporate these findings into efforts combating the obesity epidemic and its associated cardiovascular and metabolic risks. Key strategies include integrating semaglutide into health care frameworks via value-based approaches, such as clinical pathways and cardiometabolic clinics, advocating for insurance coverage, and leveraging scale to negotiate lower drug costs at scale.

## Notes

Supplement.
Data Sharing Statement

## References

[1] Powell-Wiley TM, Poirier P, Burke LE, ; American Heart Association Council on Lifestyle and Cardiometabolic Health; Council on Cardiovascular and Stroke Nursing; Council on Clinical Cardiology; Council on Epidemiology and Prevention; and Stroke Council. Obesity and cardiovascular disease: a scientific statement from the American Heart Association. Circulation. 2021;143(21):e984-e1010. doi:10.1161/CIR.0000000000000973 33882682 PMC8493650

[2] Lincoff AM, Brown-Frandsen K, Colhoun HM, ; SELECT Trial Investigators. Semaglutide and cardiovascular outcomes in obesity without diabetes. N Engl J Med. 2023;389(24):2221-2232. doi:10.1056/NEJMoa2307563 37952131

[3] Parcha V, Kalra R, Suri SS, . Geographic variation in cardiovascular health among American adults. Mayo Clin Proc. 2021;96(7):1770-1781. doi:10.1016/j.mayocp.2020.12.034 33775420 PMC8260439

[4] US Census Bureau (Population Division). Annual Estimates of the Resident Population for the United States, Regions, States, District of Columbia, and Puerto Rico: April 1, 2020 to July 1, 2023 (NST-EST2023-POP). Revised December 18, 2023. Accessed February 1, 2024. https://www.census.gov/data/tables/time-series/demo/popest/2020s-state-total.html

[5] Center for Disease Control and Prevention. Weighting Calculated Variables in the 2021 Data File of the Behavioral Risk Factor Surveillance System. Published online 2021. Accessed May 1, 2023. https://www.cdc.gov/brfss/annual_data/2021/pdf/2021-weighting-description-508.pdf

[6] Lu Y, Liu Y, Jastreboff AM, . Eligibility for cardiovascular risk reduction therapy in the United States based on SELECT trial criteria: insights from the National Health and Nutrition Examination Survey. Circ Cardiovasc Qual Outcomes. 2024;17(1):e010640. doi:10.1161/CIRCOUTCOMES.123.010640 37950677 PMC10782930

