# Supplementary material for: National and State Estimates of SELECT Trial Eligibility and Its Public Health Impact in the US
Source: JAMA Netw Open. 2024 Jul 3;7(7):e2420105. doi: 10.1001/jamanetworkopen.2024.20105 (PMC11222994; doi:10.1001/jamanetworkopen.2024.20105)
Supplement: Supplement. — Data Sharing Statement [file jamanetwopen-e2420105-s001.pdf]

Data Sharing Statement

Erhabor. National and State Estimates of SELECT Trial Eligibility and Its Public Health Impact in the US. *JAMA Netw Open*. Published July 03, 2024.
doi:10.1001/jamanetworkopen.2024.20105

Data
Data available: Yes
Data types: Deidentified participant data
How to access data: BRFSS
When available: With publication

Supporting Documents
Document types: None

Additional Information
Who can access the data: N/A
Types of analyses: N/A
Mechanisms of data availability: The data from the Behavioral Risk Factor Surveillance System (BRFSS) is available online.
